# Aqua­(2,9-dimethyl-1,10-phenanthroline-κ^2^ *N*,*N*′)bis­(2-hydroxy­benzoato-κ*O*)manganese(II) 2,9-dimethyl-1,10-phenanthroline hemisolvate

**DOI:** 10.1107/S1600536809000981

**Published:** 2009-01-14

**Authors:** Pei-Zheng Zhao, Feng-Mei Yan, Jian-Ge Wang

**Affiliations:** a College of Chemistry and Environmental Science, Henan Normal University, Xinxiang 453007, People’s Republic of China; b Department of Chemistry and Chemical Engineering, Huanghuai University, Zhumadian 463000, People’s Republic of China; c Department of Chemistry, Luoyang Normal University, Luoyang 471022, People’s Republic of China

## Abstract

In the asymmetric unit of the title complex, [Mn(C_7_H_5_O_3_)_2_(C_14_H_12_N_2_)(H_2_O)]·0.5C_14_H_12_N_2_, the Mn^II^ ion is coordinated by a bidentate 2,9-dimethyl-1,10-phenanthroline (dmphen) mol­ecule, one water mol­ecule and two monodentate 2-hydroxy­benzoate anions in a distorted trigonal-bipyramidal geometry. The OH group of the 2-hydroxy­benzoate anion is disordered over two positions with site-occupancy factors of 0.5. The asymmetric unit is completed with by an uncoordinated half-mol­ecule of dmphen, disordered about a crystallographic twofold axis. In the crystal structure, mol­ecules are linked into a two-dimensional framework by O—H⋯N, O—H⋯O and C—H⋯O hydrogen bonds. The packing of the structure is further stabilized by π–π stacking inter­actions involving dmphen mol­ecules, with centroid–centroid separations of 3.8027 (3) and 3.6319 (3) Å.

## Related literature

For background to Mn- and phenanthroline-containing complexes, see: Rüttinger & Dismukes (1997 ▶); Wang *et al.* (1996 ▶); Wall *et al.* (1999 ▶); Naing *et al.* (1995 ▶). For related structures, see: Shen & Yuan (2004 ▶); Pan & Xu (2005 ▶); Su *et al.* (2005 ▶); Pan *et al.* (2006 ▶); Shen *et al.* (2007 ▶); Xuan *et al.* (2007 ▶); Zhao *et al.* (2007 ▶).
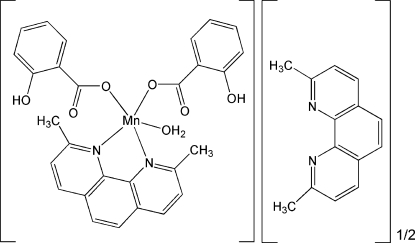


## Experimental

### 

#### Crystal data

[Mn(C_7_H_5_O_3_)_2_(C_14_H_12_N_2_)(H_2_O)]·0.5C_14_H_12_N_2_
*M* *_r_* = 659.56
Monoclinic, 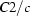

*a* = 23.225 (2) Å
*b* = 19.6902 (17) Å
*c* = 14.0225 (12) Å
β = 94.342 (1)°
*V* = 6394.2 (10) Å^3^
*Z* = 8
Mo *K*α radiation
μ = 0.47 mm^−1^
*T* = 293 (2) K
0.49 × 0.43 × 0.36 mm

#### Data collection

Bruker SMART CCD area-detector diffractometer
Absorption correction: multi-scan (*SADABS*; Bruker, 2004 ▶) *T*_min_ = 0.804, *T*_max_ = 0.849
23566 measured reflections
5959 independent reflections
4384 reflections with *I* > 2σ(*I*)
*R*_int_ = 0.022

#### Refinement

*R*[*F* ^2^ > 2σ(*F* ^2^)] = 0.050
*wR*(*F* ^2^) = 0.157
*S* = 1.02
5959 reflections
449 parameters
152 restraints
H-atom parameters constrained
Δρ_max_ = 0.34 e Å^−3^
Δρ_min_ = −0.33 e Å^−3^

### 

Data collection: *SMART* (Bruker, 2004 ▶); cell refinement: *SAINT* (Bruker, 2004 ▶); data reduction: *SAINT*; program(s) used to solve structure: *SHELXS97* (Sheldrick, 2008 ▶); program(s) used to refine structure: *SHELXL97* (Sheldrick, 2008 ▶); molecular graphics: *SHELXTL* (Sheldrick, 2008 ▶); software used to prepare material for publication: *publCIF* (Westrip, 2009 ▶).

## Supplementary Material

Crystal structure: contains datablocks I, global. DOI: 10.1107/S1600536809000981/bh2207sup1.cif

Structure factors: contains datablocks I. DOI: 10.1107/S1600536809000981/bh2207Isup2.hkl

Additional supplementary materials:  crystallographic information; 3D view; checkCIF report

## Figures and Tables

**Table:** 

Mn1—O5	2.105 (3)
Mn1—O1	2.108 (2)
Mn1—O8	2.135 (3)
Mn1—N2	2.252 (2)
Mn1—N1	2.262 (2)

**Table:** 

O1—Mn1—N2	169.01 (9)
O5—Mn1—O8	119.39 (14)
O5—Mn1—N1	127.02 (11)
O8—Mn1—N1	110.00 (12)

**Table 2:** Hydrogen-bond geometry (Å, °)

*D*—H⋯*A*	*D*—H	H⋯*A*	*D*⋯*A*	*D*—H⋯*A*
O8—H2*W*⋯N3′	0.83	2.52	3.083 (5)	127
O8—H2*W*⋯N3	0.83	2.23	3.026 (5)	160
O8—H1*W*⋯O2	0.82	1.79	2.571 (3)	159
O7—H7⋯O6	0.82	1.88	2.609 (6)	147
O4—H4*D*⋯O2	0.82	1.82	2.453 (7)	133
O3—H3*D*⋯O1	0.82	1.79	2.514 (5)	146
C12—H12*C*⋯O8	0.96	2.50	3.309 (5)	142
O8—H2*W*⋯N3^i^	0.83	2.38	3.070 (4)	141
O8—H2*W*⋯N3′^i^	0.83	2.30	3.013 (6)	145
C6—H6⋯O6^ii^	0.93	2.57	3.450 (5)	158

Symmetry codes: (i) 
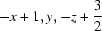
; (ii) 
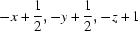
.

**Fig. 1.**
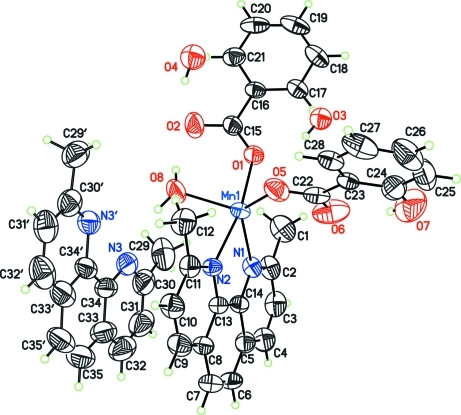
The molecular structure of the title complex with atom labels and 30% probability displacement ellipsoids for non-H atoms.

**Fig. 2.**
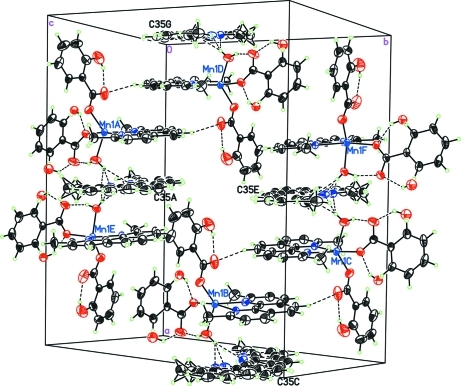
The hydrogen bonds (dashed lines) in the crystal structure of (I). Displacement ellipsoids are at the 20% probability level.

**Fig. 3.**
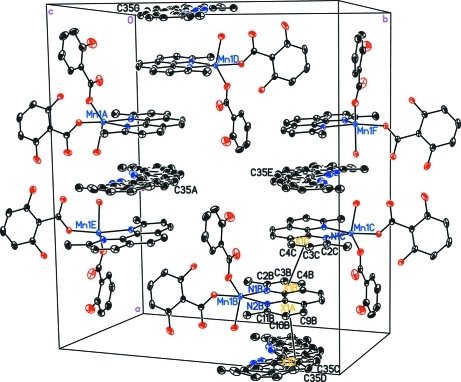
The π–π interactions between the dmphen rings of neighboring molecules in the crystal structure of (I), with 10% probability displacement ellipsoids. H atoms have been omitted for clarity. symmetry codes: (Mn1B, C35C) x + 1/2, y + 1/2, z; (Mn1C, C35E) -x + 1, -y + 1, -z + 1; (Mn1D, C35G) -x + 1/2, -y + 1/2, -z + 1; (Mn1E) -x + 1, y, -z + 3/2; (Mn1F) x, -y + 1, z - 1/2.

## supplementary crystallographic information

### Comment

It is generally believed that manganese plays an important role in biological systems (Rüttinger & Dismukes, 1997). In addition, metal-phenanthroline complexes and their derivatives have attracted much attention during recent decades because of their peculiar features (Wang *et al.*, 1996; Wall *et al.*, 1999; Naing *et al.*, 1995). A number of Mn(II) complexes have been synthesized and structures determined (Shen & Yuan, 2004; Pan & Xu, 2005; Su *et al.*, 2005; Pan *et al.*, 2006; Shen *et al.*, 2007; Xuan *et al.*, 2007; Zhao *et al.*, 2007). The title complex, (I), was recently obtained from the reaction of manganese nitrate, sodium 2-hydroxybenzoate and dmphen in an ethanol/water mixture, and its crystal structure is reported here.

The structure of the title compound, (I), is shown in Fig. 1. The Mn^II^ ion is five-coordinated by two N atoms from a dmphen ligand, and three O atoms from two 2-hydroxybenzoate ligands and a water molecule. The [MnO_3_N_2_] unit presents a distorted trigonal bipyramidal geometry, with N2 and O1 atoms occupying the axial positions, with axial O1—Mn1—N2 angle being 169.01 (9)°. The corresponding bond lengths are listed in Table 1. The OH group in one 2-hydroxybenzoate ligand is disordered over two positions with equal site occupancy factors. The whole uncoordinated dmphen molecule present in the asymmetric unit is also disordered equally between two sites related by a twofold axis.

The intramolecular hydrogen bonds between the hydroxy group, water molecule and uncoordinated carboxyl O atoms stabilize the conformation of the complex. In the crystal structure, molecules are linked into a two-dimensional framework by O—H···N and C—H···O hydrogen bonds (Table 2, Fig. 2). A partially overlapped arrangement of neighboring parallel Mn1B-dmphen (symmetry code: *x* + 1/2, *y* + 1/2, *z*) and Mn1C-dmphen rings (symmetry code: -*x* + 1, -*y* + 1, -*z* + 1) is observed in the crystal structure (Fig. 3). The shorter face-to-face separation of 3.3894 (16) Å clearly indicates the existence of π–π stacking interactions between the dmphen ligands. Furthermore, the distance between the ring centroids X1A (C8B···C11B/N2B/C13B) of coordinated Mn1B-dmphen and X1D (C33C···C35C/C33D···C35D) of uncoordinated C35C-dmphen (symmetry code: *x* + 1/2, *y* + 1/2, *z*) is 3.6319 (3) Å. This value is identical to the van der Waals thickness of the π–π stacking interaction between the nearly parallel coordinated dmphen and uncoordinated dmphen [dihedral angle: 1.36 (6)°], although dmphen rings are well overlapped with respect to each other (Fig. 3). This combination of hydrogen bonds and π–π stacking interactions builds a three-dimensional network architecture in the crystal.

### Experimental

2-hydroxybenzoic acid (0.0697 g, 0.5 mmol) and NaOH (0.0194 g, 0.5 mmol) were dissolved in distilled water (10 ml) and a 50% solution of Mn(NO_3_)_2_ (0.2103 g, 0.5 mmol) was added. This solution was added to a solution of 2,9-dimethyl-1,10-phenanthroline hemihydrate (C_14_H_12_N_2_.0.5H_2_O, 0.1089 g, 0.5 mmol) in ethanol (10 ml). The mixture was stirred at 323 K and then refluxed for 5 h, cooled to room temperature and filtered. Yellow single crystals of (I) appeared over a period of 8 d. by slow evaporation at room temperature.

### Refinement

The OH group of a 2-hydroxybenzoate anion is disordered over two positions and site occupancy factors were fixed to 1/2. The whole uncoordinated dmphen is also disordered by symmetry, and its occupation factor in the asymmetric unit was fixed to 1/2. For this dmphen molecule (16 non-H atoms), displacement parameters were restrained: a rigid bond restraint was applied to connected atoms [DELU (Sheldrick, 2008)] and bonded atoms were restrained to have the same *U*_ij_ components [SIMU (Sheldrick, 2008)]. Methyl H and hydroxyl H atoms were placed in calculated positions, with C—H = 0.96 and O—H = 0.82 Å, and refined with free torsion angles to fit the electron density; *U*_iso_(H) = 1.5*U*_eq_(carrier atom). Other H atoms were placed in calculated positions, with C—H = 0.93 Å, and refined using the riding-model approximation with *U*_iso_(H) = 1.2*U*_eq_(carrier C).

### Crystal data

**Table:**

[Mn(C_7_H_5_O_3_)_2_(C_14_H_12_N_2_)(H_2_O)]·0.5C_14_H_12_N_2_	*F*(000) = 2736
*M**_r_* = 659.56	*D*_x_ = 1.370 Mg m^−^^3^
Monoclinic, *C*2/*c*	Mo *K*α radiation, λ = 0.71073 Å
Hall symbol: -C 2yc	Cell parameters from 7104 reflections
*a* = 23.225 (2) Å	θ = 2.5–23.7°
*b* = 19.6902 (17) Å	µ = 0.47 mm^−^^1^
*c* = 14.0225 (12) Å	*T* = 293 K
β = 94.342 (1)°	Block, yellow
*V* = 6394.2 (10) Å^3^	0.49 × 0.43 × 0.36 mm
*Z* = 8	

### Data collection

**Table:**

Bruker SMART CCD area-detector diffractometer	5959 independent reflections
Radiation source: fine-focus sealed tube	4384 reflections with *I* > 2σ(*I*)
graphite	*R*_int_ = 0.022
φ and ω scans	θ_max_ = 25.5°, θ_min_ = 2.5°
Absorption correction: multi-scan (*SADABS*; Bruker, 2004)	*h* = −28→28
*T*_min_ = 0.804, *T*_max_ = 0.849	*k* = −23→23
23566 measured reflections	*l* = −16→16

### Refinement

**Table:**

Refinement on *F*^2^	Primary atom site location: structure-invariant direct methods
Least-squares matrix: full	Secondary atom site location: difference Fourier map
*R*[*F*^2^ > 2σ(*F*^2^)] = 0.050	Hydrogen site location: inferred from neighbouring sites
*wR*(*F*^2^) = 0.157	H-atom parameters constrained
*S* = 1.02	*w* = 1/[σ^2^(*F*_o_^2^) + (0.0779*P*)^2^ + 6.8361*P*] where *P* = (*F*_o_^2^ + 2*F*_c_^2^)/3
5959 reflections	(Δ/σ)_max_ = 0.001
449 parameters	Δρ_max_ = 0.34 e Å^−^^3^
152 restraints	Δρ_min_ = −0.33 e Å^−^^3^
0 constraints	

### Fractional atomic coordinates and isotropic or equivalent isotropic displacement parameters (Å^2^)

**Table:**

	*x*	*y*	*z*	*U*_iso_*/*U*_eq_	Occ. (<1)
Mn1	0.336181 (19)	0.09320 (2)	0.70559 (3)	0.05897 (18)	
O1	0.34065 (10)	−0.01168 (11)	0.6768 (2)	0.0831 (7)	
O2	0.43061 (12)	−0.04509 (14)	0.7166 (3)	0.1290 (13)	
O5	0.27211 (13)	0.07614 (13)	0.8014 (2)	0.0973 (8)	
O6	0.21336 (19)	0.07237 (18)	0.6732 (2)	0.1386 (14)	
O7	0.1036 (2)	0.0548 (3)	0.6931 (3)	0.1689 (17)	
H7	0.1328	0.0607	0.6647	0.253*	
O8	0.42499 (11)	0.08236 (12)	0.7546 (3)	0.1120 (11)	
H1W	0.4357	0.0437	0.7435	0.168*	
H2W	0.4477	0.1137	0.7451	0.168*	
N1	0.32803 (11)	0.15690 (14)	0.57097 (16)	0.0647 (6)	
N2	0.34711 (10)	0.20176 (11)	0.75410 (16)	0.0563 (6)	
C1	0.3151 (2)	0.0612 (2)	0.4651 (3)	0.1138 (15)	
H1A	0.3500	0.0402	0.4915	0.171*	
H1B	0.3102	0.0521	0.3977	0.171*	
H1C	0.2828	0.0431	0.4957	0.171*	
C2	0.31853 (16)	0.1361 (2)	0.4812 (2)	0.0828 (10)	
C3	0.31289 (17)	0.1831 (3)	0.4035 (2)	0.0924 (12)	
H3A	0.3073	0.1674	0.3409	0.111*	
C4	0.31578 (18)	0.2507 (2)	0.4215 (3)	0.0972 (12)	
H4A	0.3113	0.2813	0.3709	0.117*	
C5	0.32514 (15)	0.2746 (2)	0.5132 (2)	0.0789 (9)	
C6	0.32921 (17)	0.34444 (19)	0.5369 (3)	0.0901 (11)	
H6	0.3247	0.3763	0.4879	0.108*	
C7	0.33927 (18)	0.36627 (19)	0.6262 (3)	0.0951 (12)	
H7A	0.3418	0.4126	0.6385	0.114*	
C8	0.34610 (14)	0.31949 (16)	0.7028 (3)	0.0744 (9)	
C9	0.35821 (17)	0.3382 (2)	0.7977 (3)	0.0914 (11)	
H9	0.3618	0.3840	0.8132	0.110*	
C10	0.36477 (17)	0.2919 (2)	0.8670 (3)	0.0868 (11)	
H10	0.3734	0.3054	0.9300	0.104*	
C11	0.35866 (14)	0.22276 (17)	0.8445 (2)	0.0685 (8)	
C12	0.36389 (18)	0.1703 (2)	0.9209 (2)	0.0926 (12)	
H12A	0.3262	0.1540	0.9330	0.139*	
H12B	0.3820	0.1898	0.9783	0.139*	
H12C	0.3869	0.1332	0.9007	0.139*	
C13	0.34150 (12)	0.24857 (14)	0.6833 (2)	0.0598 (7)	
C14	0.33147 (13)	0.22509 (15)	0.5877 (2)	0.0622 (7)	
C15	0.37894 (15)	−0.05680 (16)	0.6906 (3)	0.0752 (9)	
C16	0.36092 (14)	−0.12862 (14)	0.6736 (2)	0.0616 (7)	
C17	0.30455 (15)	−0.14487 (16)	0.6432 (2)	0.0712 (8)	
H17	0.2770	−0.1109	0.6325	0.085*	0.50
O4	0.4564 (3)	−0.1659 (4)	0.7233 (5)	0.1179 (19)*	0.50
H4D	0.4645	−0.1278	0.7047	0.177*	0.50
C18	0.2891 (2)	−0.2119 (2)	0.6277 (3)	0.0975 (13)	
H18	0.2514	−0.2228	0.6063	0.117*	
C19	0.3292 (3)	−0.2622 (2)	0.6438 (3)	0.1088 (16)	
H19	0.3182	−0.3071	0.6334	0.131*	
C20	0.3841 (3)	−0.24798 (19)	0.6744 (3)	0.1033 (14)	
H20	0.4107	−0.2830	0.6849	0.124*	
C21	0.40101 (18)	−0.18138 (17)	0.6903 (3)	0.0817 (10)	
H21	0.4388	−0.1714	0.7123	0.098*	0.50
O3	0.2628 (2)	−0.0972 (3)	0.6327 (4)	0.0854 (13)*	0.50
H3D	0.2762	−0.0602	0.6491	0.128*	0.50
C22	0.22287 (19)	0.06925 (16)	0.7612 (3)	0.0768 (9)	
C23	0.17424 (14)	0.05748 (13)	0.8236 (2)	0.0645 (8)	
C24	0.1176 (2)	0.0519 (2)	0.7857 (4)	0.1016 (13)	
C25	0.0734 (2)	0.0432 (3)	0.8492 (6)	0.132 (2)	
H25	0.0351	0.0397	0.8253	0.158*	
C26	0.0871 (3)	0.0399 (3)	0.9424 (6)	0.137 (2)	
H26	0.0575	0.0349	0.9831	0.165*	
C27	0.1427 (3)	0.0436 (2)	0.9813 (4)	0.1140 (17)	
H27	0.1509	0.0395	1.0470	0.137*	
C28	0.18578 (16)	0.05344 (15)	0.9226 (2)	0.0727 (9)	
H28	0.2236	0.0575	0.9488	0.087*	
C29	0.4784 (4)	0.1418 (3)	0.5219 (3)	0.161 (5)	0.50
H29A	0.5106	0.1126	0.5394	0.241*	0.50
H29B	0.4774	0.1517	0.4547	0.241*	0.50
H29C	0.4432	0.1195	0.5358	0.241*	0.50
C30	0.4845 (3)	0.2056 (3)	0.5768 (3)	0.125 (3)	0.50
C31	0.4823 (2)	0.2698 (3)	0.5390 (2)	0.145 (3)	0.50
H31	0.4767	0.2753	0.4731	0.174*	0.50
C32	0.4880 (3)	0.3258 (3)	0.5959 (3)	0.143 (3)	0.50
H32	0.4860	0.3690	0.5688	0.172*	0.50
C33	0.4969 (2)	0.31839 (18)	0.6938 (3)	0.124 (2)	0.50
C34	0.49936 (18)	0.25172 (16)	0.7319 (2)	0.0965 (18)	0.50
C35	0.5041 (3)	0.37274 (15)	0.7589 (4)	0.143 (3)	0.50
H35	0.5024	0.4168	0.7349	0.171*	0.50
N3	0.4929 (2)	0.1958 (2)	0.6702 (3)	0.0967 (19)	0.50
C29'	0.5197 (4)	0.0964 (2)	0.9930 (4)	0.124 (3)	0.50
H29D	0.5221	0.0680	0.9378	0.186*	0.50
H29E	0.4851	0.0859	1.0234	0.186*	0.50
H29F	0.5527	0.0884	1.0371	0.186*	0.50
C30'	0.5187 (2)	0.1684 (2)	0.9634 (3)	0.104 (2)	0.50
C31'	0.52517 (19)	0.2231 (3)	1.0247 (2)	0.115 (2)	0.50
H31'	0.5307	0.2159	1.0903	0.139*	0.50
C32'	0.5236 (2)	0.2876 (2)	0.9901 (3)	0.131 (3)	0.50
H32'	0.5281	0.3240	1.0322	0.158*	0.50
C33'	0.5152 (2)	0.29941 (17)	0.8935 (3)	0.114 (2)	0.50
C34'	0.50877 (17)	0.24249 (15)	0.8322 (2)	0.0898 (19)	0.50
C35'	0.5130 (3)	0.36443 (15)	0.8510 (3)	0.131 (3)	0.50
H35'	0.5180	0.4025	0.8900	0.157*	0.50
N3'	0.5108 (2)	0.17597 (16)	0.8701 (3)	0.0930 (19)	0.50

### Atomic displacement parameters (Å^2^)

**Table:**

	*U*^11^	*U*^22^	*U*^33^	*U*^12^	*U*^13^	*U*^23^
Mn1	0.0702 (3)	0.0426 (2)	0.0647 (3)	−0.00241 (19)	0.0088 (2)	0.00082 (18)
O1	0.0740 (14)	0.0444 (11)	0.129 (2)	0.0033 (10)	−0.0054 (13)	−0.0120 (12)
O2	0.0761 (17)	0.0733 (18)	0.231 (4)	0.0028 (13)	−0.030 (2)	−0.038 (2)
O5	0.0916 (19)	0.0795 (17)	0.125 (2)	−0.0098 (14)	0.0375 (17)	0.0049 (15)
O6	0.223 (4)	0.126 (3)	0.0712 (19)	0.038 (3)	0.038 (2)	0.0291 (17)
O7	0.183 (4)	0.187 (4)	0.125 (3)	0.009 (4)	−0.066 (3)	−0.005 (3)
O8	0.0700 (15)	0.0613 (14)	0.202 (3)	−0.0088 (11)	−0.0083 (18)	−0.0295 (17)
N1	0.0730 (16)	0.0756 (17)	0.0456 (13)	−0.0130 (13)	0.0055 (11)	−0.0043 (11)
N2	0.0652 (14)	0.0512 (13)	0.0524 (13)	0.0012 (10)	0.0040 (10)	−0.0015 (10)
C1	0.141 (4)	0.116 (3)	0.086 (3)	−0.021 (3)	0.012 (3)	−0.041 (3)
C2	0.087 (2)	0.102 (3)	0.0592 (19)	−0.017 (2)	0.0081 (16)	−0.0144 (18)
C3	0.097 (3)	0.135 (4)	0.0450 (18)	−0.010 (3)	0.0034 (17)	−0.001 (2)
C4	0.103 (3)	0.121 (4)	0.067 (2)	−0.014 (3)	0.002 (2)	0.023 (2)
C5	0.075 (2)	0.092 (3)	0.069 (2)	−0.0074 (18)	0.0006 (16)	0.0210 (18)
C6	0.095 (3)	0.064 (2)	0.110 (3)	0.0003 (19)	0.003 (2)	0.037 (2)
C7	0.107 (3)	0.056 (2)	0.121 (3)	0.0011 (19)	−0.001 (3)	0.014 (2)
C8	0.074 (2)	0.0536 (18)	0.096 (3)	0.0012 (15)	0.0059 (18)	0.0041 (17)
C9	0.099 (3)	0.067 (2)	0.107 (3)	0.0013 (19)	0.004 (2)	−0.028 (2)
C10	0.098 (3)	0.080 (2)	0.082 (2)	−0.002 (2)	0.005 (2)	−0.031 (2)
C11	0.0689 (19)	0.072 (2)	0.0648 (19)	0.0012 (15)	0.0073 (14)	−0.0141 (15)
C12	0.110 (3)	0.117 (3)	0.0497 (18)	−0.001 (2)	−0.0023 (18)	−0.0007 (19)
C13	0.0626 (17)	0.0503 (15)	0.0661 (18)	0.0010 (13)	0.0027 (13)	0.0063 (13)
C14	0.0654 (18)	0.0583 (17)	0.0629 (18)	−0.0053 (14)	0.0034 (14)	0.0127 (14)
C15	0.075 (2)	0.0547 (18)	0.095 (2)	−0.0048 (16)	0.0003 (18)	−0.0109 (16)
C16	0.084 (2)	0.0441 (15)	0.0564 (16)	−0.0013 (14)	0.0062 (15)	−0.0026 (12)
C17	0.087 (2)	0.0599 (18)	0.0681 (19)	−0.0136 (16)	0.0133 (16)	−0.0041 (15)
C18	0.118 (3)	0.071 (2)	0.107 (3)	−0.037 (2)	0.026 (2)	−0.016 (2)
C19	0.178 (5)	0.050 (2)	0.102 (3)	−0.026 (3)	0.038 (3)	−0.006 (2)
C20	0.163 (5)	0.050 (2)	0.098 (3)	0.022 (2)	0.020 (3)	0.0111 (19)
C21	0.104 (3)	0.062 (2)	0.078 (2)	0.0064 (18)	−0.0002 (19)	−0.0021 (16)
C22	0.110 (3)	0.0456 (16)	0.077 (2)	0.0060 (18)	0.024 (2)	0.0095 (15)
C23	0.078 (2)	0.0359 (14)	0.079 (2)	−0.0028 (13)	0.0055 (16)	−0.0021 (13)
C24	0.106 (3)	0.079 (3)	0.116 (3)	−0.002 (2)	−0.020 (3)	−0.014 (2)
C25	0.083 (3)	0.112 (4)	0.202 (6)	−0.022 (3)	0.017 (4)	−0.034 (4)
C26	0.127 (5)	0.091 (3)	0.204 (7)	−0.024 (3)	0.078 (5)	−0.037 (4)
C27	0.178 (5)	0.072 (3)	0.099 (3)	−0.018 (3)	0.061 (3)	−0.014 (2)
C28	0.099 (2)	0.0497 (17)	0.072 (2)	−0.0065 (16)	0.0232 (18)	−0.0021 (14)
C29	0.127 (10)	0.212 (10)	0.141 (9)	−0.047 (10)	−0.005 (9)	−0.002 (8)
C30	0.086 (5)	0.157 (6)	0.134 (6)	−0.016 (6)	0.007 (5)	0.050 (5)
C31	0.103 (6)	0.176 (7)	0.157 (7)	0.002 (7)	0.009 (6)	0.069 (5)
C32	0.105 (6)	0.146 (7)	0.180 (7)	0.013 (6)	0.023 (6)	0.067 (5)
C33	0.093 (4)	0.097 (4)	0.185 (6)	0.013 (4)	0.022 (6)	0.041 (4)
C34	0.065 (3)	0.079 (3)	0.148 (5)	0.006 (5)	0.020 (4)	0.030 (3)
C35	0.111 (5)	0.085 (4)	0.233 (7)	0.002 (7)	0.025 (6)	0.016 (6)
N3	0.066 (4)	0.110 (4)	0.116 (5)	−0.006 (4)	0.017 (4)	0.030 (4)
C29'	0.109 (7)	0.143 (7)	0.119 (7)	0.008 (7)	0.005 (6)	0.035 (6)
C30'	0.079 (5)	0.122 (5)	0.111 (5)	0.008 (4)	0.015 (5)	0.005 (4)
C31'	0.082 (4)	0.147 (6)	0.120 (5)	−0.008 (5)	0.018 (4)	−0.026 (4)
C32'	0.093 (5)	0.141 (5)	0.163 (6)	−0.011 (5)	0.026 (5)	−0.043 (5)
C33'	0.077 (5)	0.090 (4)	0.179 (5)	−0.001 (4)	0.032 (5)	−0.016 (4)
C34'	0.061 (4)	0.069 (3)	0.142 (5)	0.000 (3)	0.023 (4)	0.003 (3)
C35'	0.100 (5)	0.076 (4)	0.220 (7)	−0.011 (4)	0.038 (7)	−0.019 (5)
N3'	0.075 (4)	0.091 (4)	0.114 (4)	−0.002 (3)	0.015 (4)	0.005 (3)

### Geometric parameters (Å, °)

**Table:**

Mn1—O5	2.105 (3)	O4—H21	0.4385
Mn1—O1	2.108 (2)	C18—C19	1.366 (6)
Mn1—O8	2.135 (3)	C18—H18	0.9300
Mn1—N2	2.252 (2)	C19—C20	1.344 (7)
Mn1—N1	2.262 (2)	C19—H19	0.9300
O1—C15	1.262 (4)	C20—C21	1.382 (5)
O2—C15	1.249 (4)	C20—H20	0.9300
O5—C22	1.243 (5)	C21—H21	0.9300
O6—C22	1.238 (4)	O3—H17	0.4262
O7—C24	1.315 (6)	O3—H3D	0.8200
O7—H7	0.8200	C22—C23	1.499 (5)
O8—H1W	0.8200	C23—C24	1.386 (5)
O8—H2W	0.8288	C23—C28	1.396 (5)
N1—C2	1.326 (4)	C24—C25	1.418 (8)
N1—C14	1.364 (4)	C25—C26	1.323 (8)
N2—C11	1.342 (4)	C25—H25	0.9300
N2—C13	1.354 (4)	C26—C27	1.366 (8)
C1—C2	1.494 (6)	C26—H26	0.9300
C1—H1A	0.9600	C27—C28	1.357 (6)
C1—H1B	0.9600	C27—H27	0.9300
C1—H1C	0.9600	C28—H28	0.9300
C2—C3	1.428 (6)	C29—C30	1.4759
C3—C4	1.356 (6)	C29—H29A	0.9600
C3—H3A	0.9300	C29—H29B	0.9600
C4—C5	1.371 (5)	C29—H29C	0.9600
C4—H4A	0.9300	C30—N3	1.3240
C5—C6	1.415 (5)	C30—C31	1.3702
C5—C14	1.429 (4)	C31—C32	1.3611
C6—C7	1.329 (6)	C31—H31	0.9300
C6—H6	0.9300	C32—C33	1.3816
C7—C8	1.414 (5)	C32—H32	0.9300
C7—H7A	0.9300	C33—C35	1.4076
C8—C9	1.389 (5)	C33—C34	1.4164
C8—C13	1.425 (4)	C34—N3	1.4017
C9—C10	1.333 (6)	C34—C34'	1.4192
C9—H9	0.9300	C35—C35'	1.3035
C10—C11	1.402 (5)	C35—H35	0.9300
C10—H10	0.9300	C29'—C30'	1.4763
C11—C12	1.486 (5)	C29'—H29D	0.9600
C12—H12A	0.9600	C29'—H29E	0.9600
C12—H12B	0.9600	C29'—H29F	0.9600
C12—H12C	0.9600	C30'—N3'	1.3159
C13—C14	1.420 (4)	C30'—C31'	1.3801
C15—C16	1.489 (4)	C31'—C32'	1.3576
C16—C17	1.383 (4)	C31'—H31'	0.9300
C16—C21	1.403 (5)	C32'—C33'	1.3740
C17—O3	1.350 (6)	C32'—H32'	0.9300
C17—C18	1.380 (5)	C33'—C35'	1.4111
C17—H17	0.9300	C33'—C34'	1.4130
O4—C21	1.368 (7)	C34'—N3'	1.4129
O4—H4D	0.8200	C35'—H35'	0.9300
			
O1—Mn1—N2	169.01 (9)	C18—C17—H17	119.6
O5—Mn1—O8	119.39 (14)	C16—C17—H17	120.5
O5—Mn1—N1	127.02 (11)	C21—O4—H4D	109.2
O8—Mn1—N1	110.00 (12)	H4D—O4—H21	110.2
O5—Mn1—O1	90.77 (10)	C19—C18—C17	120.1 (4)
O1—Mn1—O8	84.46 (9)	C19—C18—H18	120.0
O5—Mn1—N2	91.58 (10)	C17—C18—H18	120.0
O8—Mn1—N2	85.04 (9)	C20—C19—C18	121.4 (4)
O1—Mn1—N1	112.67 (10)	C20—C19—H19	119.3
N2—Mn1—N1	74.11 (9)	C18—C19—H19	119.3
C15—O1—Mn1	134.8 (2)	C19—C20—C21	120.0 (4)
C22—O5—Mn1	113.5 (3)	C19—C20—H20	120.0
C24—O7—H7	109.5	C21—C20—H20	120.0
Mn1—O8—H1W	109.3	O4—C21—C20	121.0 (5)
Mn1—O8—H2W	119.0	O4—C21—C16	119.1 (4)
H1W—O8—H2W	117.1	C20—C21—C16	119.9 (4)
C2—N1—C14	118.0 (3)	C20—C21—H21	120.2
C2—N1—Mn1	128.2 (2)	C16—C21—H21	119.9
C14—N1—Mn1	113.69 (18)	C17—O3—H3D	109.4
C11—N2—C13	119.0 (3)	H17—O3—H3D	106.6
C11—N2—Mn1	126.0 (2)	O6—C22—O5	122.3 (4)
C13—N2—Mn1	114.98 (18)	O6—C22—C23	120.3 (4)
C2—C1—H1A	109.5	O5—C22—C23	117.4 (3)
C2—C1—H1B	109.5	C24—C23—C28	118.9 (4)
H1A—C1—H1B	109.5	C24—C23—C22	121.6 (4)
C2—C1—H1C	109.5	C28—C23—C22	119.6 (3)
H1A—C1—H1C	109.5	O7—C24—C23	122.2 (5)
H1B—C1—H1C	109.5	O7—C24—C25	119.2 (5)
N1—C2—C3	121.6 (4)	C23—C24—C25	118.6 (5)
N1—C2—C1	116.8 (3)	C26—C25—C24	119.7 (5)
C3—C2—C1	121.6 (3)	C26—C25—H25	120.1
C4—C3—C2	119.6 (3)	C24—C25—H25	120.1
C4—C3—H3A	120.2	C25—C26—C27	122.7 (5)
C2—C3—H3A	120.2	C25—C26—H26	118.6
C3—C4—C5	120.9 (4)	C27—C26—H26	118.6
C3—C4—H4A	119.5	C28—C27—C26	119.0 (5)
C5—C4—H4A	119.5	C28—C27—H27	120.5
C4—C5—C6	123.8 (4)	C26—C27—H27	120.5
C4—C5—C14	116.9 (4)	C27—C28—C23	121.1 (4)
C6—C5—C14	119.3 (3)	C27—C28—H28	119.5
C7—C6—C5	122.6 (3)	C23—C28—H28	119.5
C7—C6—H6	118.7	N3—C30—C31	121.1
C5—C6—H6	118.7	N3—C30—C29	113.1
C6—C7—C8	120.4 (4)	C31—C30—C29	125.8
C6—C7—H7A	119.8	C32—C31—C30	121.4
C8—C7—H7A	119.8	C32—C31—H31	119.3
C9—C8—C7	123.9 (4)	C30—C31—H31	119.3
C9—C8—C13	116.8 (3)	C31—C32—C33	119.8
C7—C8—C13	119.3 (3)	C31—C32—H32	120.1
C10—C9—C8	121.4 (3)	C33—C32—H32	120.1
C10—C9—H9	119.3	C32—C33—C35	124.4
C8—C9—H9	119.3	C32—C33—C34	118.1
C9—C10—C11	119.8 (3)	C35—C33—C34	117.5
C9—C10—H10	120.1	N3—C34—C33	119.8
C11—C10—H10	120.1	N3—C34—C34'	120.8
N2—C11—C10	121.4 (3)	C33—C34—C34'	119.4
N2—C11—C12	117.8 (3)	C35'—C35—C33	123.3
C10—C11—C12	120.8 (3)	C35'—C35—H35	118.4
C11—C12—H12A	109.5	C33—C35—H35	118.4
C11—C12—H12B	109.5	C30—N3—C34	119.7
H12A—C12—H12B	109.5	N3'—C30'—C31'	122.0
C11—C12—H12C	109.5	N3'—C30'—C29'	112.8
H12A—C12—H12C	109.5	C31'—C30'—C29'	125.2
H12B—C12—H12C	109.5	C32'—C31'—C30'	120.6
N2—C13—C14	118.0 (3)	C32'—C31'—H31'	119.7
N2—C13—C8	121.6 (3)	C30'—C31'—H31'	119.7
C14—C13—C8	120.3 (3)	C31'—C32'—C33'	120.6
N1—C14—C13	119.1 (2)	C31'—C32'—H32'	119.7
N1—C14—C5	123.0 (3)	C33'—C32'—H32'	119.7
C13—C14—C5	117.9 (3)	C32'—C33'—C35'	124.6
O2—C15—O1	124.4 (3)	C32'—C33'—C34'	117.7
O2—C15—C16	118.3 (3)	C35'—C33'—C34'	117.7
O1—C15—C16	117.3 (3)	N3'—C34'—C33'	120.5
C17—C16—C21	118.8 (3)	N3'—C34'—C34	119.3
C17—C16—C15	121.1 (3)	C33'—C34'—C34	120.2
C21—C16—C15	120.1 (3)	C35—C35'—C33'	122.0
O3—C17—C18	118.1 (4)	C35—C35'—H35'	119.0
O3—C17—C16	121.9 (3)	C33'—C35'—H35'	119.0
C18—C17—C16	119.9 (4)	C30'—N3'—C34'	118.5
			
O5—Mn1—O1—C15	−115.4 (4)	O1—C15—C16—C17	0.5 (5)
O8—Mn1—O1—C15	4.0 (4)	O2—C15—C16—C21	2.4 (5)
N2—Mn1—O1—C15	−13.1 (8)	O1—C15—C16—C21	−178.1 (3)
N1—Mn1—O1—C15	113.4 (4)	C21—C16—C17—O3	174.9 (4)
O1—Mn1—O5—C22	−81.1 (2)	C15—C16—C17—O3	−3.7 (5)
O8—Mn1—O5—C22	−165.2 (2)	C21—C16—C17—C18	−1.6 (5)
N2—Mn1—O5—C22	109.6 (2)	C15—C16—C17—C18	179.8 (3)
N1—Mn1—O5—C22	38.4 (3)	O3—C17—C18—C19	−175.6 (4)
O5—Mn1—N1—C2	−100.0 (3)	C16—C17—C18—C19	1.0 (6)
O1—Mn1—N1—C2	9.4 (3)	C17—C18—C19—C20	−0.2 (7)
O8—Mn1—N1—C2	101.8 (3)	C18—C19—C20—C21	0.0 (7)
N2—Mn1—N1—C2	−179.7 (3)	C19—C20—C21—O4	178.7 (5)
O5—Mn1—N1—C14	77.4 (2)	C19—C20—C21—C16	−0.6 (6)
O1—Mn1—N1—C14	−173.16 (19)	C17—C16—C21—O4	−177.9 (4)
O8—Mn1—N1—C14	−80.8 (2)	C15—C16—C21—O4	0.7 (6)
N2—Mn1—N1—C14	−2.3 (2)	C17—C16—C21—C20	1.4 (5)
O5—Mn1—N2—C11	54.5 (3)	C15—C16—C21—C20	−180.0 (3)
O1—Mn1—N2—C11	−47.8 (6)	Mn1—O5—C22—O6	−0.1 (4)
O8—Mn1—N2—C11	−64.9 (3)	Mn1—O5—C22—C23	−179.5 (2)
N1—Mn1—N2—C11	−177.3 (3)	O6—C22—C23—C24	−2.1 (5)
O5—Mn1—N2—C13	−125.9 (2)	O5—C22—C23—C24	177.4 (3)
O1—Mn1—N2—C13	131.8 (5)	O6—C22—C23—C28	179.4 (3)
O8—Mn1—N2—C13	114.7 (2)	O5—C22—C23—C28	−1.2 (4)
N1—Mn1—N2—C13	2.30 (19)	C28—C23—C24—O7	−179.5 (4)
C14—N1—C2—C3	1.0 (5)	C22—C23—C24—O7	1.9 (6)
Mn1—N1—C2—C3	178.3 (3)	C28—C23—C24—C25	0.7 (5)
C14—N1—C2—C1	179.9 (3)	C22—C23—C24—C25	−177.8 (4)
Mn1—N1—C2—C1	−2.8 (5)	O7—C24—C25—C26	179.7 (6)
N1—C2—C3—C4	−1.7 (6)	C23—C24—C25—C26	−0.5 (7)
C1—C2—C3—C4	179.4 (4)	C24—C25—C26—C27	−1.0 (9)
C2—C3—C4—C5	1.2 (6)	C25—C26—C27—C28	2.4 (8)
C3—C4—C5—C6	179.3 (4)	C26—C27—C28—C23	−2.1 (6)
C3—C4—C5—C14	−0.1 (6)	C24—C23—C28—C27	0.6 (5)
C4—C5—C6—C7	−178.8 (4)	C22—C23—C28—C27	179.2 (3)
C14—C5—C6—C7	0.6 (6)	N3—C30—C31—C32	−0.6
C5—C6—C7—C8	−0.4 (7)	C29—C30—C31—C32	179.5
C6—C7—C8—C9	178.2 (4)	C30—C31—C32—C33	0.6
C6—C7—C8—C13	−0.6 (6)	C31—C32—C33—C35	179.2
C7—C8—C9—C10	−179.3 (4)	C31—C32—C33—C34	−0.3
C13—C8—C9—C10	−0.5 (6)	C32—C33—C34—N3	0.0
C8—C9—C10—C11	−0.8 (6)	C35—C33—C34—N3	−179.5
C13—N2—C11—C10	0.3 (4)	C32—C33—C34—C34'	179.6
Mn1—N2—C11—C10	179.9 (2)	C35—C33—C34—C34'	0.1
C13—N2—C11—C12	179.3 (3)	C32—C33—C35—C35'	−179.3
Mn1—N2—C11—C12	−1.1 (4)	C34—C33—C35—C35'	0.2
C9—C10—C11—N2	1.0 (6)	C31—C30—N3—C34	0.3
C9—C10—C11—C12	−178.0 (4)	C29—C30—N3—C34	−179.8
C11—N2—C13—C14	177.6 (3)	C33—C34—N3—C30	0.0
Mn1—N2—C13—C14	−2.0 (3)	C34'—C34—N3—C30	−179.6
C11—N2—C13—C8	−1.6 (4)	N3'—C30'—C31'—C32'	0.1
Mn1—N2—C13—C8	178.7 (2)	C29'—C30'—C31'—C32'	179.9
C9—C8—C13—N2	1.7 (5)	C30'—C31'—C32'—C33'	0.2
C7—C8—C13—N2	−179.3 (3)	C31'—C32'—C33'—C35'	−180.0
C9—C8—C13—C14	−177.5 (3)	C31'—C32'—C33'—C34'	−0.3
C7—C8—C13—C14	1.4 (5)	C32'—C33'—C34'—N3'	0.1
C2—N1—C14—C13	179.9 (3)	C35'—C33'—C34'—N3'	179.8
Mn1—N1—C14—C13	2.2 (3)	C32'—C33'—C34'—C34	179.2
C2—N1—C14—C5	0.2 (5)	C35'—C33'—C34'—C34	−1.1
Mn1—N1—C14—C5	−177.5 (2)	N3—C34—C34'—N3'	−0.9
N2—C13—C14—N1	−0.1 (4)	C33—C34—C34'—N3'	179.5
C8—C13—C14—N1	179.1 (3)	N3—C34—C34'—C33'	180.0
N2—C13—C14—C5	179.5 (3)	C33—C34—C34'—C33'	0.4
C8—C13—C14—C5	−1.2 (4)	C33—C35—C35'—C33'	−1.0
C4—C5—C14—N1	−0.6 (5)	C32'—C33'—C35'—C35	−179.0
C6—C5—C14—N1	179.9 (3)	C34'—C33'—C35'—C35	1.4
C4—C5—C14—C13	179.7 (3)	C31'—C30'—N3'—C34'	−0.2
C6—C5—C14—C13	0.2 (5)	C29'—C30'—N3'—C34'	179.9
Mn1—O1—C15—O2	−10.6 (7)	C33'—C34'—N3'—C30'	0.1
Mn1—O1—C15—C16	169.9 (2)	C34—C34'—N3'—C30'	−179.0
O2—C15—C16—C17	−179.0 (4)		

### Hydrogen-bond geometry (Å, °)

**Table:**

*D*—H···*A*	*D*—H	H···*A*	*D*···*A*	*D*—H···*A*
O8—H2W···N3'	0.83	2.52	3.083 (5)	127
O8—H2W···N3	0.83	2.23	3.026 (5)	160
O8—H1W···O2	0.82	1.79	2.571 (3)	159
O7—H7···O6	0.82	1.88	2.609 (6)	147
O4—H4D···O2	0.82	1.82	2.453 (7)	133
O3—H3D···O1	0.82	1.79	2.514 (5)	146
C12—H12C···O8	0.96	2.50	3.309 (5)	142
O8—H2W···N3^i^	0.83	2.38	3.070 (4)	141
O8—H2W···N3'^i^	0.83	2.30	3.013 (6)	145
C6—H6···O6^ii^	0.93	2.57	3.450 (5)	158

Symmetry codes: (i) −*x*+1, *y*, −*z*+3/2; (ii) −*x*+1/2, −*y*+1/2, −*z*+1.
